# 2-Hydr­oxy-*N*′-[1-(3-methyl­pyrazin-2-yl)ethyl­idene]benzohydrazide

**DOI:** 10.1107/S1600536808006697

**Published:** 2008-03-14

**Authors:** Tai Xi-Shi, Feng Yi-Min

**Affiliations:** a Department of Chemistry and Chemical Engineering, Weifang University, Weifang 261061, People’s Republic of China

## Abstract

The mol­ecule of the title compound, C_14_H_14_N_4_O_2_, is slightly twisted, with a dihedral angle of 10.06 (14)° between the aromatic rings. An intra­molecular N—H⋯O and an inter­molecular O—H⋯O hydrogen bond help to establish the crystal structure.

## Related literature

For related literature, see: Tai *et al.* (2003 ▶).
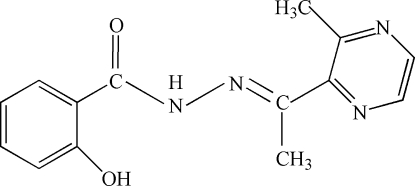


## Experimental

### 

#### Crystal data

C_14_H_14_N_4_O_2_
*M* *_r_* = 270.29
Orthorhombic, 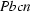

*a* = 12.6326 (14) Å
*b* = 9.3346 (10) Å
*c* = 22.119 (3) Å
*V* = 2608.3 (5) Å^3^
*Z* = 8
Mo *K*α radiation
μ = 0.10 mm^−1^
*T* = 298 (2) K
0.49 × 0.43 × 0.22 mm

#### Data collection

Bruker SMART CCD diffractometer
Absorption correction: multi-scan (*SADABS*; Bruker, 2000 ▶) *T*_min_ = 0.954, *T*_max_ = 0.979
12690 measured reflections
2303 independent reflections
1380 reflections with *I* > 2σ(*I*)
*R*_int_ = 0.052

#### Refinement

*R*[*F* ^2^ > 2σ(*F* ^2^)] = 0.044
*wR*(*F* ^2^) = 0.156
*S* = 1.07
2303 reflections
181 parameters
H-atom parameters constrained
Δρ_max_ = 0.20 e Å^−3^
Δρ_min_ = −0.17 e Å^−3^

### 

Data collection: *SMART* (Bruker, 2000 ▶); cell refinement: *SAINT* (Bruker, 2000 ▶); data reduction: *SAINT*; program(s) used to solve structure: *SHELXS97* (Sheldrick, 2008 ▶); program(s) used to refine structure: *SHELXL97* (Sheldrick, 2008 ▶); molecular graphics: *SHELXTL* (Sheldrick, 2008 ▶); software used to prepare material for publication: *SHELXTL*.

## Supplementary Material

Crystal structure: contains datablocks global, I. DOI: 10.1107/S1600536808006697/hb2708sup1.cif

Structure factors: contains datablocks I. DOI: 10.1107/S1600536808006697/hb2708Isup2.hkl

Additional supplementary materials:  crystallographic information; 3D view; checkCIF report

## Figures and Tables

**Table 1:** Hydrogen-bond geometry (Å, °)

*D*—H⋯*A*	*D*—H	H⋯*A*	*D*⋯*A*	*D*—H⋯*A*
N1—H1⋯O2	0.86	1.95	2.624 (3)	134
O2—H2⋯O1^i^	0.82	1.82	2.631 (3)	168

Symmetry code: (i) 
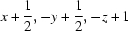
.

**Fig. 1.**
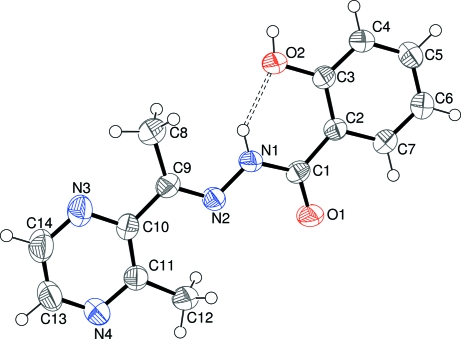
The molecular structure of (I) showing 50% displacement ellipsoids. The hydrogen bond is indicated by a double-dashed line.

## supplementary crystallographic information

### Comment

As part of our ongoing studies of the chemistry of aroylhydrazone ligands (Tai *et al.*, 2003), we now report the synthesis and structure of the title compound, (I), (Fig. 1).

The molecule is slightly twisted, with a dihedral angle of 10.06 (14)° between the aromatic rings. An intramolecular N—H···O and an intermolecular O—H···O hydrogen bond help to establish the structure (Table 1).

### Experimental

10 mmol of 2-acetyl-3-methylpyrazine (10 mmol) was added to a solution of salicyloyl hydrazine (10 mmol) in 10 ml of ethanol. The mixture was continuously stirred for 3 h at refluxing temperature, evaporating some ethanol. Upon cooling, the solid product was collected by filtration and dried *in vacuo* (yield 62%). Colourless blocks of (I) were obtained by evaporation from a methanol solution after one week.

### Refinement

The H atoms were placed geometrically (C—H = 0.93–0.96 Å, O—H = 0.82 Å, N—H = 0.86 Å) and refined as riding with *U*_iso_(H) = 1.2*U*_eq_(carrier) or 1.5*U*_eq_(methyl C).

### Crystal data

**Table:**

C_14_H_14_N_4_O_2_	*F*_000_ = 1136
*M**_r_* = 270.29	*D*_x_ = 1.377 Mg m^−^^3^
Orthorhombic, *P**b**c**n*	Mo *K*α radiation λ = 0.71073 Å
Hall symbol: -P 2n 2ab	Cell parameters from 2332 reflections
*a* = 12.6326 (14) Å	θ = 2.5–23.7º
*b* = 9.3346 (10) Å	µ = 0.10 mm^−^^1^
*c* = 22.119 (3) Å	*T* = 298 (2) K
*V* = 2608.3 (5) Å^3^	Block, colourless
*Z* = 8	0.49 × 0.43 × 0.22 mm

### Data collection

**Table:**

Bruker SMART CCD diffractometer	2303 independent reflections
Radiation source: fine-focus sealed tube	1380 reflections with *I* > 2σ(*I*)
Monochromator: graphite	*R*_int_ = 0.052
*T* = 298(2) K	θ_max_ = 25.0º
ω scans	θ_min_ = 1.8º
Absorption correction: multi-scan(SADABS; Bruker, 2000)	*h* = −15→13
*T*_min_ = 0.954, *T*_max_ = 0.979	*k* = −11→10
12690 measured reflections	*l* = −26→24

### Refinement

**Table:**

Refinement on *F*^2^	Secondary atom site location: difference Fourier map
Least-squares matrix: full	Hydrogen site location: inferred from neighbouring sites
*R*[*F*^2^ > 2σ(*F*^2^)] = 0.044	H-atom parameters constrained
*wR*(*F*^2^) = 0.156	*w* = 1/[σ^2^(*F*_o_^2^) + (0.0649*P*)^2^ + 1.2514*P*] where *P* = (*F*_o_^2^ + 2*F*_c_^2^)/3
*S* = 1.07	(Δ/σ)_max_ < 0.001
2303 reflections	Δρ_max_ = 0.20 e Å^−^^3^
181 parameters	Δρ_min_ = −0.16 e Å^−^^3^
Primary atom site location: structure-invariant direct methods	Extinction correction: none

### Special details

**Table:**

Geometry. All e.s.d.'s (except the e.s.d. in the dihedral angle between two l.s. planes) are estimated using the full covariance matrix. The cell e.s.d.'s are taken into account individually in the estimation of e.s.d.'s in distances, angles and torsion angles; correlations between e.s.d.'s in cell parameters are only used when they are defined by crystal symmetry. An approximate (isotropic) treatment of cell e.s.d.'s is used for estimating e.s.d.'s involving l.s. planes.
Refinement. Refinement of *F*^2^ against ALL reflections. The weighted *R*-factor *wR* and goodness of fit *S* are based on *F*^2^, conventional *R*-factors *R* are based on *F*, with *F* set to zero for negative *F*^2^. The threshold expression of *F*^2^ > σ(*F*^2^) is used only for calculating *R*-factors(gt) *etc*. and is not relevant to the choice of reflections for refinement. *R*-factors based on *F*^2^ are statistically about twice as large as those based on *F*, and *R*- factors based on ALL data will be even larger.

### Fractional atomic coordinates and isotropic or equivalent isotropic displacement parameters (Å^2^)

**Table:**

	*x*	*y*	*z*	*U*_iso_*/*U*_eq_	
N1	0.79886 (17)	0.1654 (2)	0.42259 (10)	0.0408 (6)	
H1	0.8658	0.1773	0.4278	0.049*	
N2	0.76327 (17)	0.0717 (2)	0.37945 (10)	0.0396 (6)	
N3	0.8738 (2)	−0.1605 (3)	0.27123 (12)	0.0590 (8)	
N4	0.6645 (2)	−0.2165 (3)	0.24385 (12)	0.0586 (8)	
O1	0.63328 (14)	0.2289 (2)	0.44838 (9)	0.0553 (6)	
O2	0.95222 (14)	0.2544 (2)	0.49322 (9)	0.0574 (6)	
H2	1.0112	0.2679	0.5076	0.086*	
C1	0.7298 (2)	0.2386 (3)	0.45667 (12)	0.0379 (7)	
C2	0.77414 (19)	0.3353 (3)	0.50398 (11)	0.0356 (6)	
C3	0.8809 (2)	0.3424 (3)	0.52082 (12)	0.0401 (7)	
C4	0.9125 (2)	0.4359 (3)	0.56555 (13)	0.0478 (8)	
H4	0.9834	0.4394	0.5767	0.057*	
C5	0.8411 (2)	0.5234 (3)	0.59369 (13)	0.0498 (8)	
H5	0.8638	0.5865	0.6235	0.060*	
C6	0.7359 (2)	0.5186 (3)	0.57807 (13)	0.0465 (8)	
H6	0.6872	0.5779	0.5973	0.056*	
C7	0.7034 (2)	0.4253 (3)	0.53383 (12)	0.0425 (7)	
H7	0.6321	0.4221	0.5235	0.051*	
C8	0.9508 (2)	0.0190 (4)	0.35800 (17)	0.0733 (11)	
H8A	0.9701	0.1181	0.3542	0.110*	
H8B	0.9873	−0.0360	0.3278	0.110*	
H8C	0.9701	−0.0150	0.3975	0.110*	
C9	0.8340 (2)	0.0033 (3)	0.34940 (13)	0.0426 (7)	
C10	0.7959 (2)	−0.0970 (3)	0.30201 (12)	0.0409 (7)	
C11	0.6890 (2)	−0.1265 (3)	0.28871 (12)	0.0428 (7)	
C12	0.5955 (2)	−0.0662 (4)	0.32156 (14)	0.0568 (9)	
H12A	0.5317	−0.1066	0.3053	0.085*	
H12B	0.5941	0.0360	0.3167	0.085*	
H12C	0.6007	−0.0892	0.3638	0.085*	
C13	0.7439 (3)	−0.2763 (4)	0.21323 (15)	0.0627 (9)	
H13	0.7283	−0.3383	0.1815	0.075*	
C14	0.8469 (3)	−0.2494 (4)	0.22696 (15)	0.0639 (10)	
H14	0.8998	−0.2945	0.2048	0.077*	

### Atomic displacement parameters (Å^2^)

**Table:**

	*U*^11^	*U*^22^	*U*^33^	*U*^12^	*U*^13^	*U*^23^
N1	0.0318 (12)	0.0478 (15)	0.0428 (14)	−0.0032 (11)	−0.0015 (10)	−0.0074 (12)
N2	0.0381 (13)	0.0423 (14)	0.0382 (13)	−0.0022 (11)	0.0012 (10)	−0.0040 (11)
N3	0.0574 (17)	0.0631 (18)	0.0565 (17)	0.0068 (14)	0.0117 (13)	−0.0102 (15)
N4	0.0638 (18)	0.0608 (18)	0.0510 (16)	0.0056 (15)	−0.0092 (13)	−0.0135 (14)
O1	0.0312 (11)	0.0761 (16)	0.0585 (13)	−0.0021 (10)	−0.0003 (9)	−0.0172 (12)
O2	0.0328 (11)	0.0712 (15)	0.0684 (14)	0.0027 (10)	−0.0054 (9)	−0.0268 (12)
C1	0.0308 (15)	0.0439 (18)	0.0389 (15)	0.0007 (13)	0.0035 (12)	0.0038 (13)
C2	0.0336 (15)	0.0361 (16)	0.0372 (15)	−0.0018 (12)	0.0008 (11)	0.0032 (13)
C3	0.0343 (15)	0.0423 (17)	0.0437 (16)	−0.0006 (13)	0.0029 (12)	−0.0033 (14)
C4	0.0374 (16)	0.0539 (19)	0.0522 (18)	−0.0010 (15)	−0.0050 (13)	−0.0084 (16)
C5	0.0552 (19)	0.0469 (19)	0.0473 (17)	−0.0040 (15)	−0.0003 (15)	−0.0080 (15)
C6	0.0506 (18)	0.0431 (18)	0.0459 (17)	0.0047 (14)	0.0040 (14)	−0.0008 (14)
C7	0.0385 (15)	0.0449 (18)	0.0440 (16)	0.0039 (14)	0.0017 (13)	0.0027 (15)
C8	0.0386 (18)	0.087 (3)	0.094 (3)	0.0031 (18)	0.0004 (17)	−0.033 (2)
C9	0.0359 (15)	0.0455 (18)	0.0464 (17)	0.0021 (14)	0.0031 (13)	0.0001 (14)
C10	0.0453 (17)	0.0388 (17)	0.0386 (15)	0.0063 (13)	0.0041 (13)	0.0013 (13)
C11	0.0474 (17)	0.0437 (18)	0.0374 (16)	0.0061 (14)	−0.0021 (13)	0.0019 (14)
C12	0.0408 (17)	0.072 (2)	0.0576 (19)	0.0035 (16)	−0.0044 (14)	−0.0143 (17)
C13	0.078 (2)	0.061 (2)	0.0490 (19)	0.011 (2)	−0.0054 (18)	−0.0167 (17)
C14	0.068 (2)	0.069 (2)	0.055 (2)	0.013 (2)	0.0114 (17)	−0.0118 (19)

### Geometric parameters (Å, °)

**Table:**

N1—C1	1.340 (3)	C5—H5	0.9300
N1—N2	1.370 (3)	C6—C7	1.373 (4)
N1—H1	0.8600	C6—H6	0.9300
N2—C9	1.284 (3)	C7—H7	0.9300
N3—C14	1.328 (4)	C8—C9	1.495 (4)
N3—C10	1.335 (3)	C8—H8A	0.9600
N4—C13	1.332 (4)	C8—H8B	0.9600
N4—C11	1.336 (4)	C8—H8C	0.9600
O1—C1	1.236 (3)	C9—C10	1.485 (4)
O2—C3	1.364 (3)	C10—C11	1.409 (4)
O2—H2	0.8200	C11—C12	1.497 (4)
C1—C2	1.492 (4)	C12—H12A	0.9600
C2—C7	1.393 (4)	C12—H12B	0.9600
C2—C3	1.400 (3)	C12—H12C	0.9600
C3—C4	1.378 (4)	C13—C14	1.359 (5)
C4—C5	1.367 (4)	C13—H13	0.9300
C4—H4	0.9300	C14—H14	0.9300
C5—C6	1.374 (4)		
			
C1—N1—N2	120.2 (2)	C9—C8—H8A	109.5
C1—N1—H1	119.9	C9—C8—H8B	109.5
N2—N1—H1	119.9	H8A—C8—H8B	109.5
C9—N2—N1	116.7 (2)	C9—C8—H8C	109.5
C14—N3—C10	117.7 (3)	H8A—C8—H8C	109.5
C13—N4—C11	117.8 (3)	H8B—C8—H8C	109.5
C3—O2—H2	109.5	N2—C9—C10	117.0 (2)
O1—C1—N1	121.5 (3)	N2—C9—C8	124.9 (3)
O1—C1—C2	121.2 (2)	C10—C9—C8	118.1 (2)
N1—C1—C2	117.3 (2)	N3—C10—C11	120.8 (3)
C7—C2—C3	117.6 (2)	N3—C10—C9	113.7 (3)
C7—C2—C1	117.2 (2)	C11—C10—C9	125.5 (2)
C3—C2—C1	125.2 (2)	N4—C11—C10	120.0 (3)
O2—C3—C4	120.7 (2)	N4—C11—C12	114.4 (3)
O2—C3—C2	119.3 (2)	C10—C11—C12	125.5 (3)
C4—C3—C2	120.0 (3)	C11—C12—H12A	109.5
C5—C4—C3	120.9 (3)	C11—C12—H12B	109.5
C5—C4—H4	119.6	H12A—C12—H12B	109.5
C3—C4—H4	119.6	C11—C12—H12C	109.5
C4—C5—C6	120.3 (3)	H12A—C12—H12C	109.5
C4—C5—H5	119.9	H12B—C12—H12C	109.5
C6—C5—H5	119.9	N4—C13—C14	121.9 (3)
C7—C6—C5	119.3 (3)	N4—C13—H13	119.0
C7—C6—H6	120.3	C14—C13—H13	119.0
C5—C6—H6	120.3	N3—C14—C13	121.7 (3)
C6—C7—C2	121.9 (3)	N3—C14—H14	119.2
C6—C7—H7	119.1	C13—C14—H14	119.2
C2—C7—H7	119.1		
			
C1—N1—N2—C9	−178.6 (3)	N1—N2—C9—C10	−179.2 (2)
N2—N1—C1—O1	−3.4 (4)	N1—N2—C9—C8	−0.2 (4)
N2—N1—C1—C2	178.2 (2)	C14—N3—C10—C11	1.6 (4)
O1—C1—C2—C7	−6.7 (4)	C14—N3—C10—C9	−178.4 (3)
N1—C1—C2—C7	171.7 (2)	N2—C9—C10—N3	177.8 (3)
O1—C1—C2—C3	173.1 (3)	C8—C9—C10—N3	−1.3 (4)
N1—C1—C2—C3	−8.6 (4)	N2—C9—C10—C11	−2.2 (4)
C7—C2—C3—O2	179.0 (2)	C8—C9—C10—C11	178.7 (3)
C1—C2—C3—O2	−0.7 (4)	C13—N4—C11—C10	0.4 (4)
C7—C2—C3—C4	0.1 (4)	C13—N4—C11—C12	−178.9 (3)
C1—C2—C3—C4	−179.6 (3)	N3—C10—C11—N4	−1.6 (4)
O2—C3—C4—C5	−179.4 (3)	C9—C10—C11—N4	178.4 (3)
C2—C3—C4—C5	−0.6 (4)	N3—C10—C11—C12	177.6 (3)
C3—C4—C5—C6	0.6 (5)	C9—C10—C11—C12	−2.4 (5)
C4—C5—C6—C7	−0.2 (4)	C11—N4—C13—C14	0.8 (5)
C5—C6—C7—C2	−0.2 (4)	C10—N3—C14—C13	−0.4 (5)
C3—C2—C7—C6	0.3 (4)	N4—C13—C14—N3	−0.9 (6)
C1—C2—C7—C6	−180.0 (3)		

### Hydrogen-bond geometry (Å, °)

**Table:**

*D*—H···*A*	*D*—H	H···*A*	*D*···*A*	*D*—H···*A*
N1—H1···O2	0.86	1.95	2.624 (3)	134
O2—H2···O1^i^	0.82	1.82	2.631 (3)	168

Symmetry codes: (i) *x*+1/2, −*y*+1/2, −*z*+1.
